# Traditional Chinese Medicine for Coronary Artery Disease Treatment: Clinical Evidence From Randomized Controlled Trials

**DOI:** 10.3389/fcvm.2021.702110

**Published:** 2021-08-06

**Authors:** Bo Liang, Ning Gu

**Affiliations:** ^1^Nanjing University of Chinese Medicine, Nanjing, China; ^2^Nanjing Hospital of Chinese Medicine Affiliated to Nanjing University of Chinese Medicine, Nanjing, China

**Keywords:** traditional Chinese medicine, coronary artery disease, review, randomized controlled trial, complementary and alternative medicine

## Abstract

Traditional Chinese medicine has a history of more than 2,000 years and has been widely used in clinical practice. However, due to the lack of a reliable scientific basis, the role of traditional Chinese medicine in the prevention and treatment of coronary artery disease is not clear. At present, the existing randomized controlled trials about traditional Chinese medicine for coronary artery disease have defects, small sample sizes, and different results, so it is difficult to make a clear conclusion on the actual advantages and disadvantages of traditional Chinese medicine. In this review, the efficacy and safety of traditional Chinese medicine in the prevention and treatment of coronary artery disease were systematically evaluated through randomized controlled trials, most of which were double-blind trials. We reviewed 17 randomized controlled trials that included a total of 11,726 coronary artery disease patients. The methodological quality of the trials was generally high, with nine (52.94%) having a modified Jadad score of 7 and only three (17.65%) having a modified Jadad score of <3. There are 16 trials (94.12%) reporting safety; the safety of traditional Chinese medicine seems not to be inferior to that of mimetic, placebo, or western medications. Moreover, the results from 17 randomized controlled trials (100.00%) showed that traditional Chinese medicine can be applied as a complementary and alternative method to the primary and secondary prevention of coronary artery disease, and only six trials (35.29%) described adverse cardiovascular events specifically. However, it is necessary to assess the safety and efficacy of traditional Chinese medicine in treating coronary artery disease with long-term hard endpoints.

## Introduction

In recent years, due to various reasons, such as an aging population, the number of cardiovascular diseases has risen sharply (1–3). Mortality related to cardiovascular diseases is also rapidly escalating worldwide and brings a great threat to global health (4). Either incidence or mortality of coronary artery disease (CAD) accounts for a large part of cardiovascular diseases (5–7). In the United States, CAD accounts for more than two-thirds of all cardiovascular diseases, and almost half of Americans have at least one risk factor (8). In Europe, cardiovascular mortality was higher in middle-income countries where it accounted for a greater proportion of potential years of life lost compared with high-income countries (9). Situations in Asian countries are not better than in western countries (10). Most countries in Asia are experiencing the challenges from CAD, with the mortality rate varying from 103 to 366 per 100,000 adult populations (11). In the clinic, CAD is often mainly divided into stable CAD (SCAD, namely, chronic myocardial ischemia syndrome) and acute coronary syndrome according to the latest clinical practice guidelines, and the treatment is mainly based on lifestyle management, medical treatment (including conventional western medications and Chinese medicine), and revascularization (12). Despite the use of medical treatment and revascularization, there is still much room for improving efficacy and reducing adverse reactions (13).

Traditional Chinese medicine (TCM), mainly from the East, has shown its idiographic ascendancy in the prevention, therapeutic effect, rehabilitation, and healthcare of diverse diseases (14–16). The evidence-based application of TCM keeps a foothold in China and other Asian countries, and with the popularity of TCM in the East, it is increasingly accepted and used by other countries in the world (17, 18). TCM doctors often prescribe a personalized mixture of herbs, characterizing holistic view and syndrome differentiation, to a patient (16). Thus, TCM opens new perspectives for the discovery and development of relevant drugs since it is a more systemic approach to preventing and treating diseases (16), resulting in a rethinking of the importance of TCM (19). So far, more and more randomized controlled trials (RCTs), which are taken to be the gold standard of testing efficacy and safety of therapies for a wide spectrum of diseases and for informing therapeutic guidelines (20), about TCM for CAD have been issued their results. These reliable results not only spur the modernization of TCM but also bring us stronger confidence in the management of CAD. However, we have to admit that RCTs of TCM were carried out relatively late, and these RCTs use surrogate endpoints, have small sample sizes, short follow-up, and diverse outcomes. Here, we critically evaluate the efficacy and safety of TCM in patients with CAD with available evidence from RCTs.

## Search Strategy and Selection Criteria

Two reviewers (BL and NG) searched PubMed, comprising more than 32 million citations for biomedical literature from MEDLINE, Life Science journals, and online books, through the National Center for Biotechnology Information, U.S. National Library of Medicine, for particular RCTs comparing the efficacy and safety of TCM in patients with CAD with language limited to English.

Inclusion criteria for RCTs: (1) Participants with definite diagnoses of CAD, including stable angina, unstable angina, myocardial infarct (MI), SCAD, and coronary artery bypass grafting, made with accepted western medicine methods. (2) The efficacy and/or safety of TCM were assessed. The assessment indicators included, but were not limited to, hard endpoints and surrogate endpoints, such as Chinese medicine syndrome, 6-min walking test, ultrasound echocardiography, serum markers, hemodynamic examination, side effects, and cardiovascular mortality. All clinical indicators were compared with no intervention or placebo, or western medications.

Exclusion criteria for RCTs: (1) The sample size was <50. (2) The follow-up duration was <2 weeks. (3) RCTs only reported symptomatic changes without any objective measurements.

When studies or data duplication were found, only the latest or most complete studies were included. We assessed the trials' methodological quality using the modified Jadad score scale. Two reviewers (BL and NG) extracted the data and assessed quality independently. Any disagreements were resolved by discussion. Extracted information was summarized on standardized reporting forms (Table 1). Although we only included RCTs, which means methodological heterogeneity is acceptable, the clinical heterogeneity is so substantial that we cannot aim to perform a meta-analysis pooling the results.

**Table 1:** Details of major randomized controlled trials.

**References**	**Patients**	**Sample size**	**Intervention**	**Comparisons**	**Outcomes**	**Follow-up**	**Efficacy**	**Safety (adverse events)**	**Blinding**	**Modified Jadad score**
Zhang et al. (21)	Stable angina (blood stasis syndrome)	240	Wufuxinnaoqing Soft Capsule	Mimetic	Angina, Chinese medicine syndrome, the withdrawal or reduce rate of nitroglycerin	12 weeks	Positive	Yes	Double	2 + 2 + 2 + 1 = 7
Xu et al. (22)	Angina pectoris after percutaneous coronary intervention	187	Shenzhu Guanxin Recipe	Placebo	Angina pectoris score, Chinese medicine symptom score, and Seattle angina questionnaire score, the death rate, restenosis, and other emergency treatments	12 months	Positive	Yes	Double	2 + 2 + 2 + 1 = 7
Li et al. (23)	Stable angina	287	Guanxinshutong Capsule	Placebo	The primary outcome in this study is the change from baseline in angina attack frequency at 4 weeks of treatment. The secondary outcomes include: (1) reduction of nitroglycerin dose; (2) score of Seattle angina questionnaire; (3) positive exercise tolerance text	4 weeks	Positive	Yes	Double	2 + 2 + 2 + 1 = 7
Zhai et al. (24)	Stable angina	65	Shengjie Tongyu Granule	Tongxinluo Capsule	Angina score and traditional Chinese medicine score	4 weeks	Positive	Yes	Not applicable	2 + 1 + 0 + 1 = 4
Wang et al. (25)	Unstable angina	60	Xin'anning Nasal Drop	Control	The changes of ST segment in resting electrocardiogram and total ischemia burden in 24-h dynamic electrocardiogram	4 weeks	Positive	Yes	Not applicable	2 + 1 + 0 + 1 = 4
Chu et al. (26)	Unstable angina with blood stasis syndrome after percutaneous coronary intervention	90	Xuefu Zhuyu Capsule	Shengmai Capsule and placebo	The clinical symptoms and signs, electrocardiography, and blood stasis syndrome scores. Short-form 36 and Seattle angina questionnaire score.	4 weeks	Positive	Yes	Double	2 + 2 + 2 + 1 = 7
Sun et al. (27)	Unstable angina	72	Danhong Injection	Blood-producing needle	Homocysteine, high-sensitivity C-reactive protein, and N-terminal pro-brain natriuretic peptide	2 weeks	Positive	Yes	Single	1 + 0 + 1 + 1 = 3
Lu et al. (28)	Previous myocardial infarction	4,870	Xuezhikang	Placebo	Major coronary event, total cardiovascular mortality, total all-cause mortality, need for coronary revascularization, and change in lipoprotein lipids	4.5 years	Positive	Yes	Double	1 + 0 + 1 + 1 = 3
Shang et al. (29)	Myocardial infarction	3,505	Qi-Shen-Yi-Qi Dripping Pill	Aspirin	The primary endpoint was a composite of cardiovascular death, non-fatal reinfarction, and non-fatal stroke. The secondary outcomes were the events of serious cardiac arrhythmias, heart failure, cardiac shock, revascularization, pulmonary embolism, and deep-vein thrombosis	18 months	Positive	Yes	Double	2 + 2 + 2 + 1 = 7
Zhang et al. (30)	ST segment elevation myocardial infarction undergoing emergency percutaneous coronary intervention	219	Tongxinluo	Placebo	Myocardial no-flow and the infarct area	6 months	Positive	Yes	Double	2 + 2 + 2 + 1 = 7
Mao et al. (31)	Acute myocardial infarction	83	Danlou Tablet	Placebo	Left ventricular volumes	90 days	Positive	Yes	Double	2 + 1 + 2 + 1 = 6
Li et al. (23)	Stable coronary artery disease	1,500	Qing-Xin-Jie-Yu Granule	Placebo	The primary outcome was a composite of cardiovascular death, non-fatal myocardial infarction, and coronary revascularization	12 months	Positive	Yes	Double	2 + 2 + 2 + 1 = 7
Zhao et al. (32)	Coronary artery disease	150	Zhibitai 480 mg + atorvastatin 10 mg	Atorvastatin (20 and 40 mg)	Lipid profile, cardiotrophin-1, and C-reactive protein	8 weeks	Positive	Yes	Not applicable	2 + 2 + 0 + 1 = 5
Wang et al. (33)	Stable coronary artery disease	114	Yugengtongyu Granule	Placebo	Major outcomes (any occurrence of cardiovascular death, non-fatal myocardial infarction, or coronary revascularization), minor outcomes (any occurrence of all-cause death, ischemic stroke, readmission due to unstable angina, heart failure, or malignant arrhythmia), and composite outcomes (union of major and minor outcomes)	18 months	Positive	Yes	Double	2 + 2 + 2 + 1 = 7
Xu et al. (34)	Coronary artery disease	58	Huoxin Formula	Control	Serum biomarkers, and cardiovascular indicators of the common and internal carotid arteries	3 months	Positive	Yes	Not applicable	1 + 0 + 0 + 1 = 2
Ma et al. (35)	Coronary artery disease following percutaneous coronary intervention and depression or anxiety	60	Xinkeshu Tablet	Placebo	Depressive/anxiety symptoms and the levels of 440 peripheral blood cytokines	12 weeks	Positive	Not applicable	Double	2 + 2 + 2 + 1 = 7
Zhang et al. (36)	Coronary artery disease received coronary artery bypass grafting	166	Shemmai	Control	6-min walking test	30 days	Positive	Yes	Single	2 + 1 + 0 + 1 = 4

## Stable Angina

Stable angina is a common symptom of stable CAD (13). The researchers conducted an RCT to assess the efficacy and safety of Wufuxinnaoqing Soft Capsule (五福心脑清胶囊), which has been widely applied to angina for about 30 years, for chronic stable angina (in line with blood stasis syndrome in TCM). It is concluded that Wufuxinnaoqing Soft Capsule could decrease angina attacks and nitroglycerin consumption, relieve angina severity degree, and effectively reduce the blood stasis syndromes after a 12-week treatment, and it was safe during the follow-up (21). Another double-blind RCT of 187 patients with angina after the percutaneous coronary intervention was conducted to confirm the efficacy and safety of Shenzhu Guanxin Recipe (参术冠心方), and the results showed a larger reduction in angina pectoris score, TCM symptom score, and Seattle Angina Questionnaire score in patients given Shenzhu Guanxin Recipe from pretreatment to 12-month follow-up assessment. Again, the data showed that the safety of Shenzhu Guanxin Recipe was acceptable (22). A phase IV double-blind, randomized, and placebo-controlled study conducted at 12 centers in China indicated that after 4-week treatment with Guanxinshutong Capsule (冠心舒通胶囊), the quality of life was substantially improved, and the number of angina attacks and the consumption of short-acting nitrates were significantly reduced (23). Both of these studies were based on the premise of standard western medication treatment with TCM, and there was no positive control group, so the study on Shengjie Tongyu Granule (升解通瘀颗粒) was born. In this study, Tongxinluo Capsule (通心络胶囊), which is clinically recognized as the effective drug in treating angina (25, 37–39), was used as a positive control drug to study the effect of Shengjie Tongyu Granule for angina. The results showed that the angina score and TCM score of the two groups were all significantly improved after the treatment, but there was no statistical significance in comparison between groups, suggesting that Shengjie Tongyu Granule can effectively improve the clinical symptoms of patients with angina, with the curative effect similar with Tongxinluo Capsule (24).

## Unstable Angina

Unstable angina is a clinical manifestation between exertive stable angina pectoris and acute MI and sudden death. Sixty patients with unstable angina were randomly assigned to two groups to determine the efficacy of add-on therapy of Xin'anning Nasal Drop (心安宁滴鼻剂) for unstable angina, and the results indicated the rapid relief effect of Xin'anning Nasal Drop (40). Another RCT divided a total of 90 unstable angina with blood stasis syndrome in TCM after successful percutaneous coronary intervention population into three groups [Xuefu Zhuyu Capsule (血府逐瘀胶囊) group, Shengmai Capsule (生脉胶囊) group, and placebo group]. After 4 weeks of treatment with corresponding medications, patients in the Xuefu Zhuyu Capsule group had better efficacy on clinical symptoms and signs and blood stasis syndrome scores, and health-related quality of life in those populations compared with those in other groups (26). Sun and his colleagues conducted an RCT that included 72 patients with unstable angina to demonstrate that the treatment of Danhong Injection (丹红注射液) for 2 weeks can reduce homocysteine, high sensitivity C-reactive protein, and N-terminal pro-brain natriuretic peptide of patients with unstable angina (27).

## Myocardial Infarct

Nearly 5,000 Chinese patients who experienced a previous MI and average low-density lipoprotein cholesterol levels at baseline were assigned to placebo to Xuezhikang (血脂康) to test the effects of Xuezhikang on lipoprotein and cardiovascular endpoints. After a 4.5-year therapy, Xuezhikang significantly decreased the recurrence of coronary events (including non-fatal MI and death of CAD) and the occurrence of new cardiovascular events as well as total mortality, improved lipoprotein regulation; at the same time, Xuezhikang was safe and well-tolerated (28). Another large sample study aimed to evaluate the effectiveness and safety of Qi-Shen-Yi-Qi Dripping Pill (芪参益气滴丸) for the secondary prevention of MI. After treatment of Qi-Shen-Yi-Qi Dripping Pill or for 1 year, the 1- and 1.5-year estimated incidences of the primary outcome (a composite of cardiovascular death, non-fatal reinfarction, and non-fatal stroke) were 2.98 and 3.67%, respectively, in the Qi-Shen-Yi-Qi Dripping Pill group and 2.96 and 3.81% in the aspirin group, meaning that the secondary prevention effect of Qi-Shen-Yi-Qi Dripping Pill on MI is similar to that of aspirin although inconclusive (29). A total of 219 patients undergoing emergency percutaneous coronary intervention for acute ST-segment elevation MI were consecutively enrolled in an RCT to evaluate the efficacy of Tongxinluo (通心络) on no-reflow and the infarction area. The results indicated that ST-segment restorations in the Tongxinluo group at 6-, 12-, and 24-h reperfusion were more significant than those in the control group. Moreover, the incidence of no-reflow at 24 h reperfusion was also significantly reduced. The myocardial perfusion scores on the 7th and 180th days after ST-segment elevation MI in Tongxinluo group were improved significantly compared with those in the control group. The great thing is, there was no significant difference in severe adverse events between the two groups (30). A large proportion of patients with MI have ventricular remodeling later. The prevention or reversal of ventricular remodeling can significantly improve the prognosis and quality of life of those populations. Standard echocardiographic evaluation revealed that compared with placebo, Danlou Tablet (丹蒌片) treatment significantly reduced the left ventricular end-systolic and end-diastolic volume indexes in addition to improving the left ventricular ejection fraction. Notably, patients in the Danlou Tablet group had a lower incidence of the major adverse cardiovascular events (31).

## Stable Coronary Artery Disease

Despite optimal secondary preventive treatment, patients with SCAD remain at high risk of cardiovascular events. Patients (1,500) with diagnosed SCAD were assigned to Qing-Xin-Jie-Yu Granule (清心解郁颗粒) or placebo for half a year and followed up for another half a year to determine the reduced risk of cardiovascular events of Qing-Xin-Jie-Yu Granule, and results demonstrated that there was no significant difference in the primary endpoint and adverse events between the Qing-Xin-Jie-Yu Granule group and the placebo group. However, the absolute risk of the composite “hard” endpoint of cardiovascular death, non-fatal MI, and ischemic stroke was reduced by 0.99% in the Qing-Xin-Jie-Yu Granule group (41). Zhibitai (脂必泰), a Chinese patent medicine for the treatment of hyperlipidemia, can significantly reduce triglycerides, total cholesterol, low-density lipoprotein-cholesterol, and apolipoprotein B with fewer adverse events in patients with SCAD. In addition, Zhibitai released obvious anti-inflammatory effects, reducing cardiotrophin-1, and high-sensitivity C-reactive protein levels (32). Moreover, the addition of Yugengtongyu Granule (愈梗通瘀颗粒) based on current standard treatment reduced the incidence of composite outcomes, defined as any occurrence of cardiovascular death, non-fatal MI, or coronary revascularization, any occurrence of all-cause death, ischemic stroke, readmission due to unstable angina, heart failure, or malignant arrhythmia, and improved quality of life in patients with SCAD (33). Another TCM, Huoxin Formula (活心方), also reduced the high-sensitivity C-reactive protein, interleukin 18, interleukin 17, and matrix metallopeptidase 9 levels in patients with SCAD (34). Moreover, Huoxin Formula delayed the increase of intima–media thicknesses of the internal carotid artery and common carotid artery, as well as the increase in cardio-ankle vascular index (34). Most patients with SCAD have depression or anxiety symptoms, and this is associated with increased mortality (42, 43). Xinkeshu Tablet (心可舒片) can effectively address anxiety and depression symptoms of patients with SCAD via balancing the inflammatory environment—results from a double-blinded, randomized, placebo-controlled, clinical trial (35).

## Coronary Artery Bypass Grafting

Coronary artery bypass grafting is a big blow to the patients, and postoperative cardiac rehabilitation needs to be put on the agenda. Recent research showed that Shenmai approach [Shengmai Injection (参麦注射液) during hospitalization and Shengmai Capsule (参麦胶囊) during 30-day follow-up after discharge combined with cardiac rehabilitation] improved the exercise tolerance of patients who received coronary artery bypass grafting (36).

## Discussion

**Figure 1:**
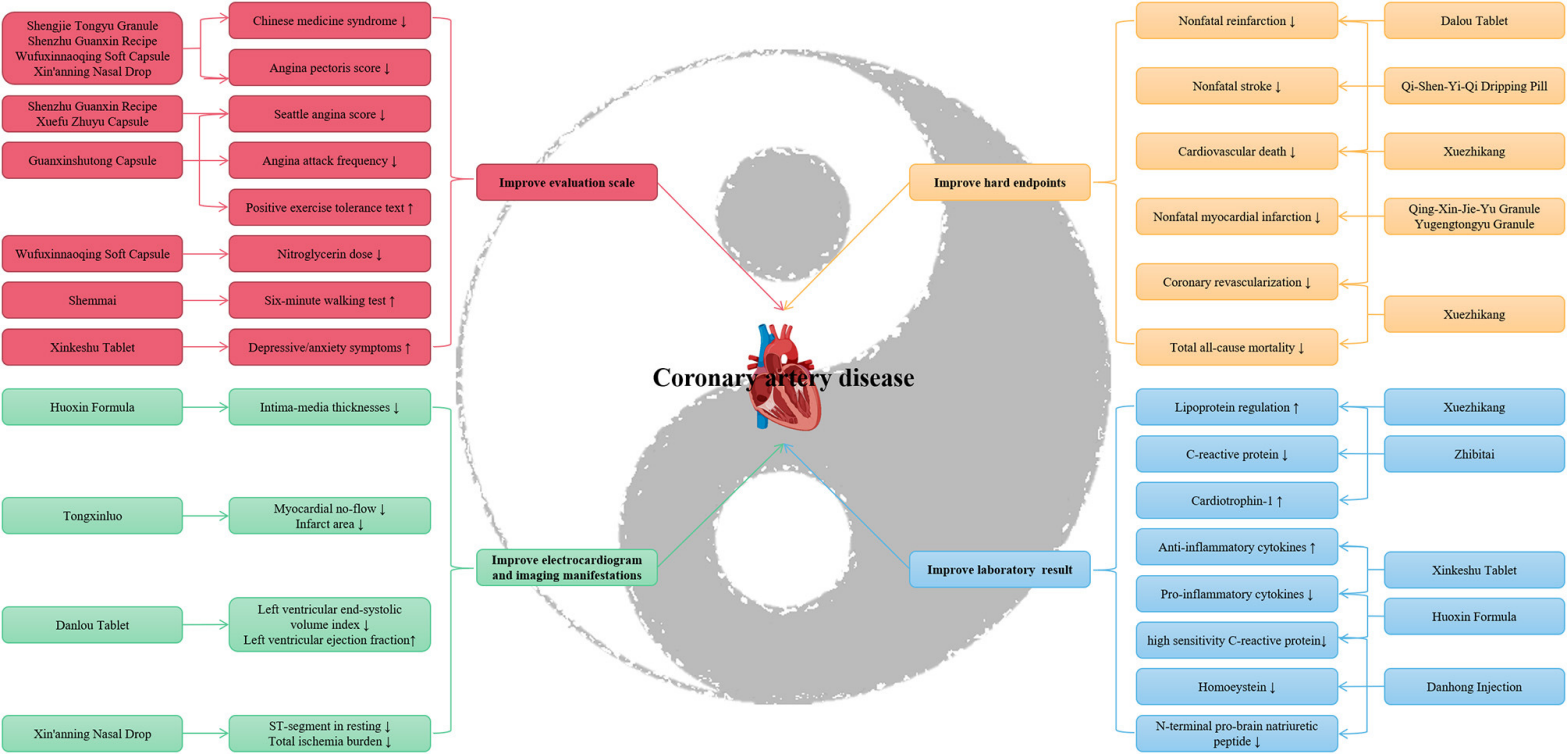
Traditional Chinese medicine in coronary artery disease protection.

TCM is a great treasure house of the Chinese nation, and its evidence-based medicine for various diseases, including CAD, is constantly being explored. Here, we systematically reviewed 17 RCTs that included a total of 11,726 patients to reappraise the efficacy and safety of TCM for CAD. The sample sizes ranged from 59 to 4,870 participants, and the mean follow-up ranged from 2 weeks to 4.5 years. We assessed the trials' quality using the modified Jadad score scale, as described previously (44), and we found that among the 17 RCTs, 9 RCTs (52.94%) got a full score of seven, and only 3 RCTs (17.65%) were identified as low-quality trials because of the less than three score (Table 1). In most RCTs, TCM was associated with significant improvements in surrogate endpoints for stable angina, unstable angina, MI, SCAD, and coronary artery bypass grafting (Table 1). However, only five trials (29.41%) described hard endpoints specifically, and only one trial (5.88%) did not report safety (Table 1). Although TCM mixture of herbs is the most commonly used in clinics (20, 45, 46), some herbs can also be prescribed solely (47). An RCT involved 100 patients with CAD demonstrated that Danshen (*Salvia miltiorrhiza Bunge*) and Gegen [*Pueraria montana var. lobata (Willd.) Maesen & S.M. Almeida ex Sanjappa & Predeep*] adjunctive treatment improved vascular function and structure effectively, and was well tolerated, suggesting they may become a novel secondary prevention approach (48). Interestingly, in addition to Danshen and Gegen, RCTs (29, 49) show that Qi-Shen-Yi-Qi Dripping Pill and Tongxinluo Capsule can also be used as a supplementary and alternative therapy for secondary prevention of MI. In general, TCM is notably effective and safe in the prevention, treatment, and health care of CAD compared with mimetic, placebo, or western medications (Table 1 and Figure 1).

Besides traditional Chinese medical (TCM) science also includes Qigong (气功) (50–52). A community-based RCT showed that Baduanjin exercise (八段锦操), a form of Qigong, increased self-efficacy in patients with SCAD, thereby increasing better self-management of the rehabilitation process (53). In addition, some other ongoing RCTs will also provide important evidence-based medicine evidence for TCM in CAD. Inflammation and immune injury in SCAD have been proven (54), and whether Taoren Honghua Jian Granule (桃仁红花煎颗粒) could alleviate symptoms, control inflammation, and improve quality of life in patients with SCAD will also be explored (55). Other RCTs will reveal the efficacy and safety of Danhong Injection, as well as its optimal timing of intervention (before or after percutaneous coronary intervention) to prevent microvascular obstruction in patients with ST-segment elevation myocardial infarction (56), and determine the clinical efficacy and safety of traditional Chinese medicine Tongxinluo Capsule in the treatment of ST-segment elevation myocardial infarction patients in the reperfusion era (57).

Our study still has some limits, which should be of concern. First, TCM pays attention to syndrome differentiation, that is to say, the first four diagnoses [Wang (望), Wen (闻), Wen (问), and Qie (切) in TCM] are combined to confirm a syndrome, and then the prescription is conducted according to the syndrome. CAD refers to thoracic obstruction (胸痹) according to TCM (20), and researches show that the common TCM syndromes of CAD are qi and blood deficiency, blood stasis, and obstruction collaterals, liver depression and spleen deficiency, and qi stagnation and blood stasis (20, 58). At present, the available RCTs do not fully consider the syndrome differentiation of TCM, which is contrary to TCM. Fortunately, many researchers are conducting RCTs to study the effect of TCM on CAD syndrome, especially qi stagnation and blood stasis syndrome) (59, 60) and qi deficiency and blood stasis syndrome (61). The results will provide clinical evidence for the application of TCM to CAD syndrome. Moreover, only English-published RCTs were evaluated, ignoring other languages (62–67), which may generate selective bias to a certain degree. Third, the comparisons are control or placebo; the combination of a positive drug and a placebo may be the most acceptable, though it is tedious (68). Moreover, here, we only evaluated the efficacy and safety efficacy of traditional Chinese medicine in stable angina, unstable angina, MI, SCAD, and coronary artery bypass grafting. Although these diseases account for the majority of CAD, they still cannot fully represent CAD. We look forward to more relevant RCTs to show the benefits of TCM on atherosclerosis. Finally, TCM has long been widely applied not only in China but also in other countries, such as Japan, Korea, and other countries. RCTs we reviewed are mainly performed in China, with small sample sizes, short follow-up, and diverse outcomes, from the hard endpoints to surrogate endpoints, rigorously designed trials are warranted.

Although TCM in the treatment of CAD has made gratifying achievements, there is still a long way to go. First, before carrying out the RCT of TCM, especially traditional Chinese medication, we should have enough syndrome differentiation information, preferably from at least three senior chief physicians, so as to be consistent with the theory of TCM and more in line with the clinical application of TCM. Second, because most traditional Chinese medication has certain color and smell, it also imperceptibly increases the difficulty of a blind method setting in clinical trials. Capsules may be a good choice. Third, in addition to the effectiveness, the safety of traditional Chinese medication is also an issue that needs attention, especially the interaction with western medication. There is still a lack of knowledge about this. In addition, although TCM has been gradually internationalized, we still need to carry out high-quality RCTs abroad to supplement the international evidence of TCM treatment for related diseases, not just CAD, and avoid race and background bias. Finally, TCM is often considered as a supplementary and alternative treatment, but there is also evidence that it can be used as primary and secondary prevention of CAD, which needs a large sample, long follow-up, and high-quality clinical trials with hard endpoint as the outcome to further verify.

In conclusion, the findings from the present study indicate that TCM may be a complementary and alternative agent for the primary and secondary prevention of patients with CAD. Thus, we recommend, at least to an extent, prescribing TCM for CAD, especially in the selected population. However, further rigorously designed trials are warranted to evaluate the effect of TCM on the long-term hard endpoints in patients with CAD.

## Author Contributions

BL and NG designed the study, acquired and researched the data for the article, and discussed its content. BL wrote the manuscript and NG revised the manuscript. All authors read and approved the final manuscript.

## Conflict of Interest

The authors declare that the research was conducted in the absence of any commercial or financial relationships that could be construed as a potential conflict of interest.

## Publisher's Note

All claims expressed in this article are solely those of the authors and do not necessarily represent those of their affiliated organizations, or those of the publisher, the editors and the reviewers. Any product that may be evaluated in this article, or claim that may be made by its manufacturer, is not guaranteed or endorsed by the publisher.
